# The effect of equine bone marrow‐derived mesenchymal stem cells on the expression of apoptotic genes in neutrophils

**DOI:** 10.1002/vms3.427

**Published:** 2021-01-20

**Authors:** Fatemeh Salami, Mitra Ghodrati, Abbas Parham, Jalil Mehrzad

**Affiliations:** ^1^ Division of Physiology Department of Basic Sciences, Veterinary Faculty Ferdowsi University of Mashhad Mashhad Iran; ^2^ Stem Cell Biology and Regenerative Medicine Research Group Research Institute of Biotechnology Ferdowsi University of Mashhad Mashhad Iran; ^3^ Department of Microbiology and Immunology Faculty of Veterinary Medicine University of Tehran Tehran Iran

**Keywords:** apoptotic genes, equine, mesenchymal stem cells, neutrophils, qPCR

## Abstract

**Background:**

Bone marrow mesenchymal stem cells (BM‐MSCs), as multipotent cells with self‐renewal and plastic‐adherent properties, have immunomodulatory effects on immune cells, including neutrophils. These cells are in close proximity in bone marrow (BM) sinusoids with non‐multiplicative immature neutrophils. BM‐MSCs exert their immunomodulatory effects on adjacent cells both directly (cell‐to‐cell contact) and indirectly (secretion of soluble factors).

**Objectives:**

The aim of this study was to evaluate the effect of equine bone marrow mesenchymal stem cells (BM‐MSCs) on the expression of some pro‐ and anti‐apoptotic genes (p53, survivin and Bcl_2_) in neutrophils co‐cultured with BM‐MSCs.

**Methods:**

For this purpose, peripheral blood neutrophils were isolated and separately co‐cultured for 12 hr with both BM‐MSCs and the BM‐MSCs΄ supernatant. Four groups were included: neutrophils with only culture media (as control), neutrophils co‐cultured with BM‐MScs, neutrophils cultured with BM‐MSCs’ supernatant and neutrophils cultured with lipopolysaccharide (LPS, as positive control). Then, the expression of mentioned genes (p53, survivin and Bcl_2_) was evaluated by quantitative polymerase chain reaction (qPCR).

**Results:**

Compared with control neutrophils, in neutrophils co‐cultured with both BM‐MSCs and BM‐MSCs’ supernatant, the mRNA expression levels of p53, as pro‐apoptotic gene, and survivin and Bcl_2_, as anti‐apoptotic genes, were remarkably increased and decreased (*p* < .05), respectively.

**Conclusions:**

These data revealed the notion that the direct contact of BM‐MSCs is not obligatory for their effects on the apoptotic status of neutrophils and they affect neutrophils via soluble secreted factors, which is promising for clinical implications in equine medicine.

## Graphical abstract

The aim of this study was to evaluate the effect of equine bone marrow mesenchymal stem cells (BM‐MSCs) on the expression of some pro‐ and anti‐apoptotic genes (p53, survivin and Bcl2) in neutrophils co‐cultured with BM‐MSCs. Results showed that BM‐MSCs or their supernatant decrease the mRNA expression of p53 (apoptotic gene) and increase the expression of survivin and Bcl2 (anti‐apoptotic genes). Our data revealed that the direct contact of BM‐MSCs or paracrine secretions support neutrophils against apoptosis, which is promising for their clinical applications in equine medicine.

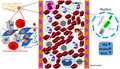

## 1 INTRODUCTION

Bone marrow mesenchymal stem cells (BM‐MSCs) are known as multipotent cells with self‐renewal and plastic‐adherent properties which are easily isolated from various sources such as bone marrow and adipose tissues. In addition to their ability to differentiate into mesodermal cell lines, the BM‐MSCs also possess immune‐modulating properties (Alipour et al., 2015; Bianco & Robey, 2000; Deans & Moseley, 2000; Zahedi et al., 2017; Zhang et al., 2009). Because of close interaction at bone marrow (BM) niche, BM‐MSCs exert an immunomodulatory effect on highly proliferating immune cells. BM‐MSCs suppress T cells activation and proliferation in vitro (Di Nicola et al., 2002) and B‐lymphocyte and NK cells function (Corcione et al., 2006; Spaggiari et al., 2008). Furthermore, BM‐MSCs secrete immunoregulatory factors and cytokines that affect immune system cells and decrease them (Kyurkchiev et al., 2014).

BM‐MSCs stimulation by pro‐inflammatory cytokines such as interferon‐γ (INF‐γ), tumour necrosis factor‐α (TNF‐α), IL‐1α or IL‐1β leads to secretion of enzymes and numerous factors causing immunosuppressive function. Induced BM‐MSCs can alter the function of different immune cells through secretion of mediators such as nitric oxide (NO), prostaglandin (PGE2), cox‐2, indoleamine 2,3‐dioxygenase (IDO) and IL‐6 (Krampera et al., 2006; Ren et al., 2008; Salami et al., 2018). It has also been shown that immunomodulation mediated by BM‐MSCs is an important defence mechanism against harmful immune activation between blood and mesenchymal stem cells in vivo (Rasmusson, 2006).

The bone marrow reserve is an available source of neutrophils with the same functional properties as their peripheral counterparts (Berkow & Dodson, 1986). Among human white blood cells, neutrophils have very short lifespan and play a key role in the innate immunity (Silva & Correia‐Neves, 2012). Apoptosis is a way whereby neutrophils maintain their balance in physiological conditions after killing pathogens (Savill et al., 2002). It has been reported that BM‐MSCs protect neutrophils from apoptosis (Raffaghello et al., 2008). This effect may be mediated by paracrine factors. Neutrophil survival is modulated through survival and death factors, however, their portion in the apoptosis regulation in vivo is unclear (Colotta et al., 1992; Lee et al., 1993; Liles, 1996). According to some studies, incubation of neutrophils with BM‐MSCs supernatant at 24 hr promotes LPS‐stimulated neutrophils’ survival, which is mainly caused by IL‐8 secretion (Brandau et al., 2010). This mechanism exerts by modulation of key mitochondrial proteins of the Bcl‐2 family, Bax and MCL‐1 (Brandau et al., 2010; Moulding et al., 1998).

P53 is a transcription factor that can directly activate the transcription of genes known to promote apoptosis (El‐Deiry, 1998; Sax & El‐Deiry, 2003; Yu et al., 1999). Survivin, named BIRC as well, is a member of the inhibitor of apoptosis (IAP) family which is involved in inhibition of apoptosis and cell cycle regulation. Overexpression of survivin prevents intrinsic and extrinsic apoptosis pathways (Altieri, 2001; Ambrosini et al., 1997; Tamm et al., 1998). Another important gene in apoptosis process is anti‐apoptotic B‐cell lymphoma‐2 (Bcl_2_), which inhibits the induction of apoptosis in malignant cells and normal cellular lineages (Opferman & Kothari, 2018).

Here, we aimed (a) to evaluate the effect of equine BM‐MSCs on the expression of p53, as a pro‐apoptotic gene, survivin and Bcl_2_, as anti‐apoptotic genes in neutrophils using qPCR method, and (b) to determine whether the effect is mediated by direct contact of BM‐MSCs with neutrophils or by their soluble factors.

## 2 MATERIALS AND METHODS

### 2.1 BM‐MSCs preparation and proliferation

Frozen fully characterized BM‐MSCs of three mares which we had previously prepared (Zahedi et al., 2017), were thawed at the second passage (P2) and cultured in high Glucose DMEM (Dulbecco's Modified Eagle Medium) (Gibco, City, USA) supplemented with 10% FBS (Fetal bovine serum), 1% penicillin/streptomycin and 0.1% amphotericin B under 37°C and 5% CO_2_ condition. The BM‐MSCs at passage 4 (P4) were seeded in 12 well plates in order to co‐culture with neutrophils.

### 2.2 Neutrophil Isolation

Blood samples were collected from external jugular vein of 6‐year‐old healthy mares (*n* = 3) as a source of neutrophils. Isolation of polymorphonuclear leukocytes (PMNs) from peripheral blood was performed using hypotonic lysis of erythrocytes according to previously described method (Mehrzad et al., 2009; Mehrzad et al., 2011; Mehrzad, Devriendt et al., 2014; Mehrzad, Maleki et al., 2014). Indeed, although the PMN isolation of equine was harder than human and cow, herein the isolation procedure routinely yielded >96% PMN with >98% (trypan blue exclusion) viability. After washing with RPMI medium, PMN suspensions were counted and adjusted to ~5×10^6^ viable PMN/ml in complete RPMI 1640 medium (R&D systems), and eventually used for BM‐MSCs‐PMNs co‐culture.

### 2.3 Experimental design

After the preparation of BM‐MSCs and neutrophils, the experimental groups were designed as follows: (a) only neutrophils as control group; (b) co‐culturing neutrophils and BM‐MSCs at a ratio of 1:35 of BM‐MSCs to neutrophils (BM‐MSCs + N) (Mehrzad, Devriendt et al., 2014; Mehrzad, Maleki et al., 2014); (c) neutrophils cultured with supernatant of BM‐MSCs as condition medium (BM‐MSCs + S) and (d) neutrophils induced by lipopolysaccharide (BM‐MSCs + LPS, as positive control). Each group contained at least five replicates. To prevent mixing of the two types of cells, BM‐MSCs at the fourth passage, 24 hr before the neutrophil extraction stage, were cultured on 12 well plates (except for the control group, 100,000 BM‐MSCs were cultured in each well). To collect the supernatant of BM‐MSCs, top fluid of cultured BM‐MScs was collected at the second day of culture. After counting by haemocytometer slide, 3.5 million neutrophils were added to each wells (1:35 BM‐MSCs:N ratio). Two ml of culture media were added to each well for control and PMN‐BM‐MSCs groups and 2 ml of the condition medium (collected from cultured BM‐MSCs) was used for wells including neutrophils (group 3). Culture time was 12 hr and, finally, neutrophils in each sample of all groups were collected for total RNA extraction.

### 2.4 Gene expression analysis

#### 2.4.1 RNA extraction and cDNA synthesis

Total RNA was extracted using Qiagen mini kit (Cat. No: 74104) according to the manufacturer's specifications. Briefly, after cell disruption, first solution (RLT) in added ethanol was added to cells. Supernatant was moved to a new tube and ethanol 70% was added to precipitate RNA and pipetting was done. Next, these samples were moved onto the column and centrifuged at 8,000*g* for 15 s. RW1 was added to the column again and centrifuged at 8,000*g* for 15 s. Then, RPE as the second solution of kit was added to the column twice to wash out ethanol residue and centrifuged at 8,000*g* for 15 s and 2 min, respectively. Finally, 50‐µl RNase‐free double‐distilled water was added to the column which was placed in a 1.5 ml microtube, and centrifuged at 8,000*g* for 1 min to wash out the extracted RNA. Concentration and quality of RNA were determined using Nano Drop spectro‐photometer (Thermo Scientific) and gel electrophoresis, respectively.

Single‐strand cDNA synthesis was performed in reverse‐transcriptase (RT‐PCR) tubes (AccuPower® RT PreMix, Cat no: K‐2041) containing 1 µg template RNA, random hexamer primer and RT Premix. The volume of the material was adjusted to 20 µl with RNase‐free double‐distilled water (ddH_2_O). The samples were centrifuged slowly and recommended thermal condition by manufacturer's description was followed. In next step, expression of GAPDH, as a housekeeping gene, was accessed to test the accuracy of molecular steps including RNA extraction, cDNA synthesis and reverse‐transcription PCR amplification in all experimental groups. In addition, the electrophoresis gel of PCR products for different genes was done to verify the correct bonding of the primers, the proliferation of the desired genes and non‐proliferation of unwanted transcripts.

#### 2.4.2 Relative quantitative real‐time PCR (qPCR)

According to the sequence information of p53, survivin, Bcl_2_ and GAPDH genes on NCBI database, specific forward and revers primers were designed using Beacon Designer 8 (Premier Biosoft International, Palo Alto, CA, USA) and primer BLAST software (Table 1). Gene expression level for p53, survivin and Bcl_2_ was analysed using quantitative real‐time PCR (qPCR) using the amplicon SYBER Green PCR kit 2x (*Cat*. *No*.: A325402) in a Fast Real‐time PCR System (Rotor Gene 6000). First, the required standard curves were obtained and efficiency of reactions was calculated for each gene. The reaction was carried out in accordance with the following temperature–time condition: First, cDNA denaturation was performed at a temperature of 94°C for 15 min. Subsequently, in 40 cycles, this temperature program was repeated: 94°C for 30 s, 61.5°C for 45 s and 72°C for 60 s. Experimental samples were run in triplicate with the same concentration of cDNA per reaction. The qPCR products were run on 1% agarose gel electrophoresis to confirm the results. Obtained threshold cycle values (CT) were also normalized to the reference gene (GAPDH) for each target gene of interest and fold change was calculated with pfaffl method (Livak & Schmittgen, 2001; Schmittgen & Livak, 2008).

**TABLE 1:** Primers characteristics used in qPCR

Gene	Accession numbers	Sequence	Annealing (°C)	Amplicon size (bp)
P53	NM_001202405.1	F: ACTATCATCACCCTGGAAGAC R:GTTACTGGACAATACTCGCTTAG	61.5	177
Survivin	XM_001915400.1	F: CGACCCCATAGAGGAACATA R: GGTTAATTCTTCAAACTGCTTCTT	61.5	79
Bcl_2_	XM_001490436.3	F: CGGAGAGTTCTAAGGATTGG R: ACTTCCTCTGTGATGTTGTATT	61.5	118
GAPDH	NM_001163856.1	F: GTCGGAGTAAACGGATTTGG R:ATGTAGTTGAGGTCAATGAAGG	61.5	113

Abbreviations: F, Forward primer; R, Reverse primer.

### 2.5 Data analysis

Ct values of all samples in treatment and control groups in each experiment (with triplicate read) were analysed by Kruskal–Wallis test using SPSS.16 software and data are presented as mean ± SD. *p* < .05 was considered as significant difference.

## 3 RESULTS

### 3.1 BM‐MSCs proliferation and co‐culture system

**FIGURE 1:**
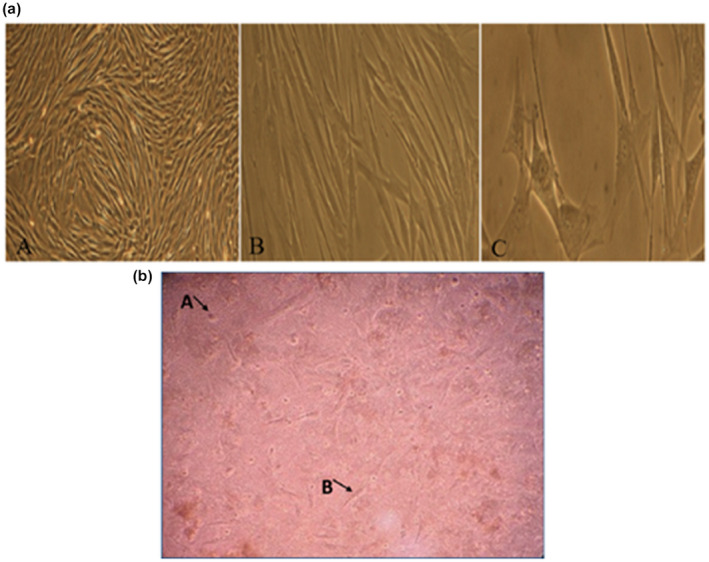
(a) Culture and proliferation of equine bone marrow‐derived mesenchymal stem cells (BM‐MSCs). Morphology of MSCs under invert microscope on Passage 3 with (a) 40×; (b) 100× and (c) 400×. (b) Co‐culture of mesenchymal stem cell (MSCs) with neutrophils. 40×. (a) a neutrophil, (b) a MSCs

The BM‐MSCs at P4 were plastic adherent and fibroblast‐like cells (Figure 1a). In co‐culture group, BM‐MSCs were attached to the bottom of culture plate and neutrophils were as suspended cells on BM‐MSCs (Figure 1b).

Only samples with proper quality and quantity were used for qPCR reactions. Absorbance values for all RNA samples at 260 and 280 nm (A260/280) and 260 and 230 nm (A260/230) were 2.0–2.2 and 1.8–2.2, respectively. Gel electrophoresis of RNA samples showed two clear bands related to rRNA (28s and 18s) along with 5s band which confirmed the quality of RNA. Expression of GAPDH, as a housekeeping gene, confirmed the accuracy of molecular steps including RNA extraction, cDNA synthesis and reverse‐transcription PCR amplification in all experimental groups. Moreover, negative control and RT minus samples confirmed no genomic DNA contamination in each experiment. In addition, the electrophoresis gel of PCR products for different genes indicated the correct bonding of the primers, the proliferation of the desired genes and non‐proliferation of unwanted genes.

**FIGURE 2:**
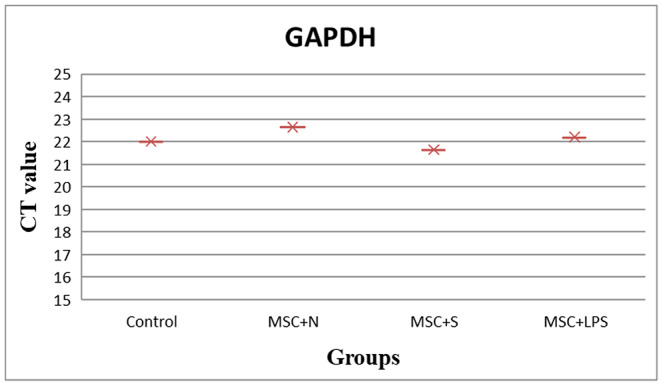
Expression pattern of reference gene (GAPDH) in different groups. Mean value of expression level (CT) show a stable expression level in all groups. BM‐MSCs, Bone marrow mesenchymal stem cells; N, neutrophils; S, supernatant; LPS, lipopolysaccharide

The expression level of reference gene (GAPDH) was almost similar in all cases which is a critical criteria for reference gene (Figure 2). The melting curve analysis showed the specificity of the GAPDH primers. In addition, primers were validated by amplification efficiencies (E = 10 − 1/slope) of 100% ± 10% and the efficiency was 0.92 for p53 and 0.99 for GAPDH, Bcl2 and survivin.

### 3.2 Expression level of anti‐apoptotic genes (Bcl‐2 and survivin)

**FIGURE 3:**
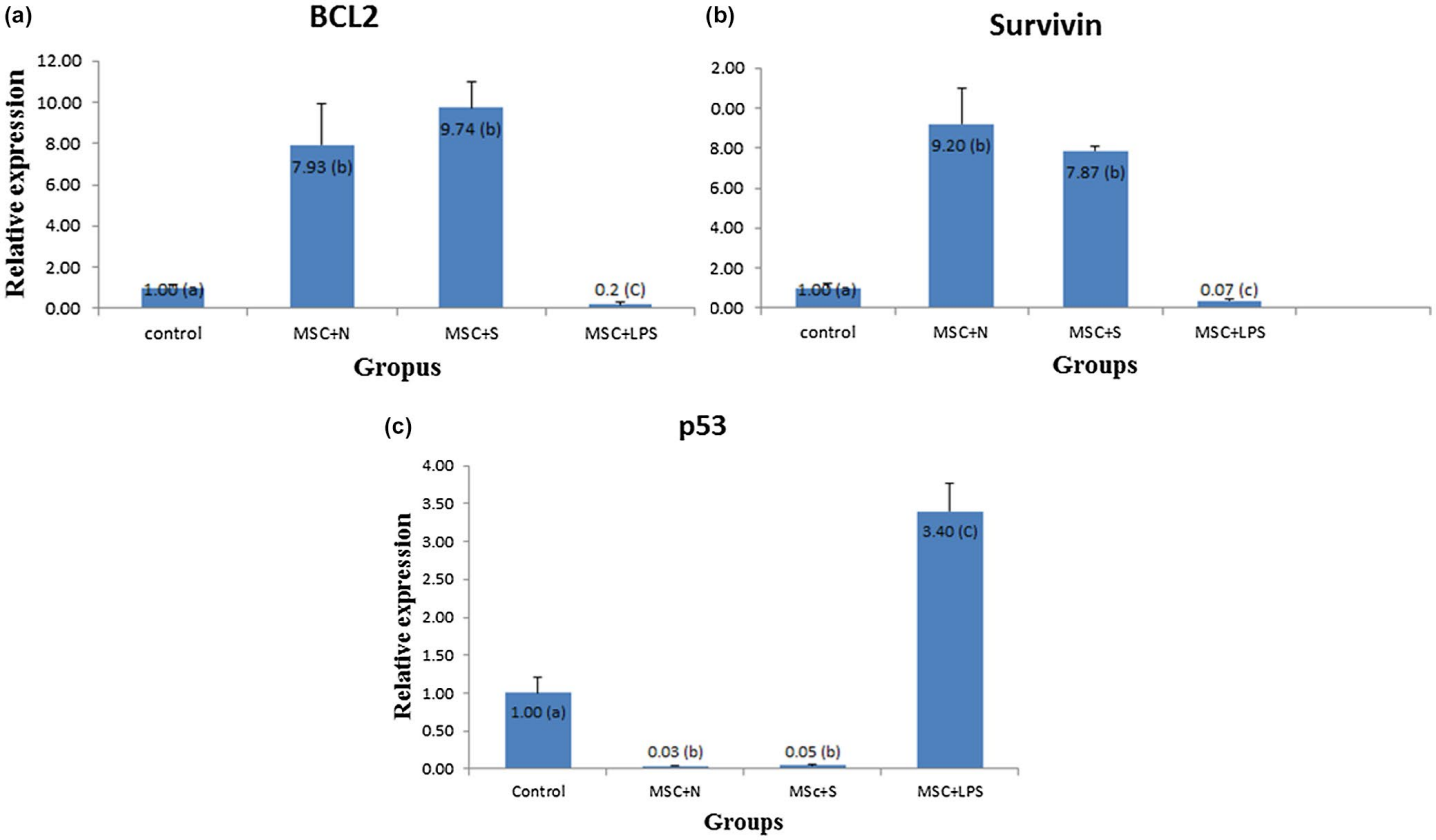
Comparison of Bcl‐2 (A) and Survivin (B) expression among different groups. Data are presented as mean ± SD. Different letters (a, b, c) show significant differences. BM‐MSCs, Bone marrow mesenchymal stem cells; N, neutrophils; S, supernatant; LPS, lipopolysaccharide. (C) Expression of P53 in treatment groups compared with control group. Data are presented as mean ± SD. Different letters (a, b, c) show significant differences. BM‐MSCs, mesenchymal stem cells; N, neutrophils; S, supernatant; LPS, lipopolysaccharide

Expression of Bcl_2_ and survivin decreased in positive control group compared to other groups (*p* < .05), which showed the induction of apoptosis in neutrophils. Significant increase in Bcl_2_ and survivin expression in BM‐MSCs + PMNs group and BM‐MSC΄s supernatant group compared to control group show that the direct contact of BM‐MSCs or their soluble factors could inhibit apoptosis process in neutrophils (Figure 3A,B).

### 3.3 Expression level of pro‐apoptotic gene (P53)

Significant increase in P53 expression in positive control group compared to others showed the apoptosis induction in neutrophils by LPS. Decreased expression of P53 in BM‐MSCs + PMNs group and BM‐MSC΄s supernatant group compared to control group (*p* < .05), showed the protective effect of BM‐MSCs and their soluble factors against apoptosis in neutrophils (Figure 3C).

Finally, the melting curve analysis showed the specificity of the P53, Bcl‐2 and surviving primers. In addition, running qPCR products on agarose gel electrophoresis confirmed the accuracy and purity of amplification products in all samples.

## 4 DISCUSSION

Many in vitro and in vivo studies have shown immunological activities of BM‐MSCs, including inhibition of T cells, B cells, killer cells and dendritic cells (Ramasamy et al., 2007). Recently, many studies have noticed the intrinsic effect of BM‐MSCs on intrinsic immunity, including their inhibitory role on active cellular proliferation and antigen delivery of macrophages and dendritic cells (Maqbool et al., 2011).

**FIGURE 4:**
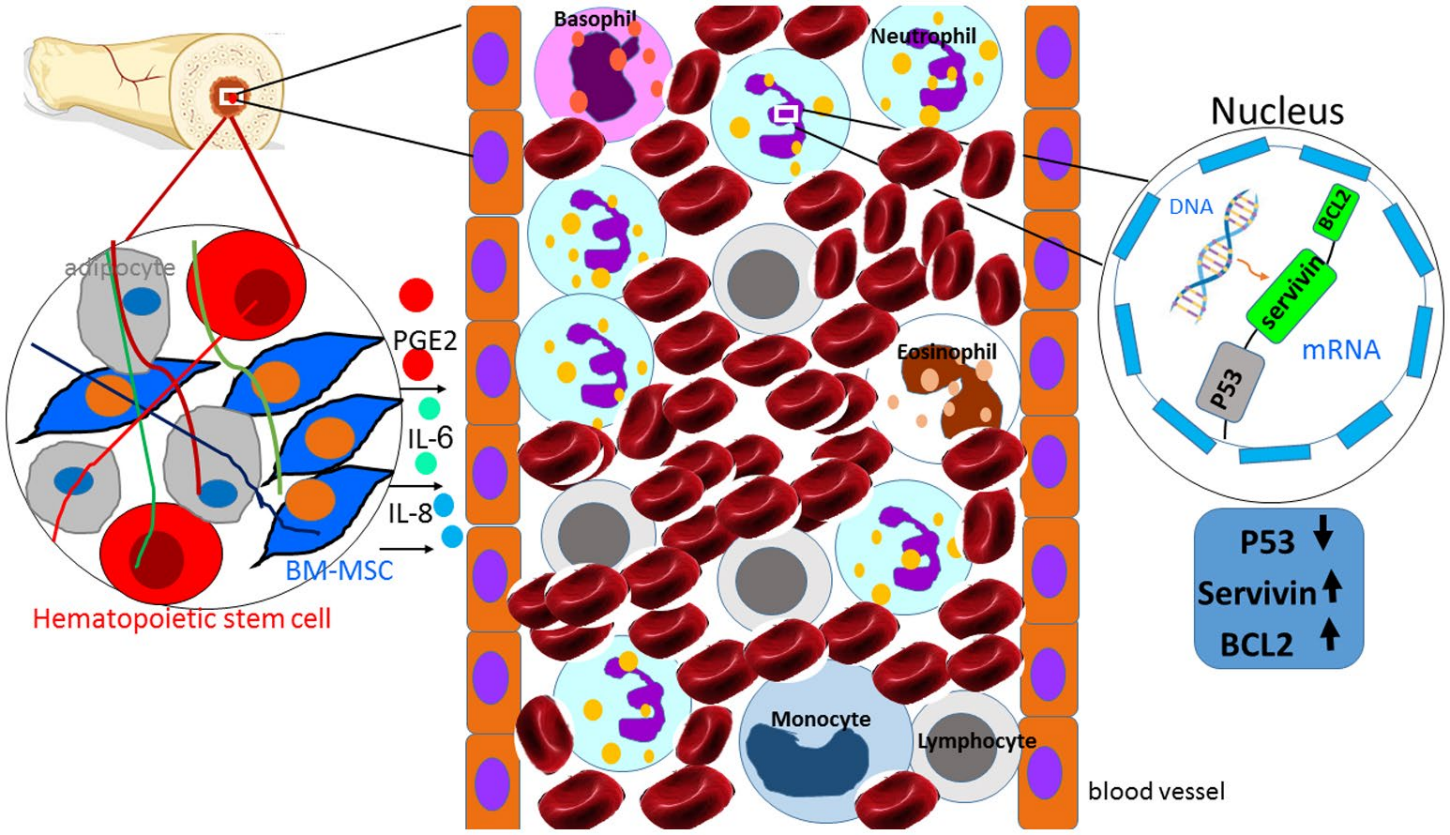
Schematic concepts of bone marrow mesenchymal stem cells (BM‐MSCs) functions on neutrophils. BM‐MSCs secrete soluble factors such as IL‐8, IL‐6 and PGE2, and affect on adjacent neutrophils in the bone marrow cavity (Nemeth & Mezey, 2015; Salami et al., 2018). For example, IL‐8 increases expression of anti‐apoptotic genes such as survivin and Bcl2, and decrease expression of pro‐apoptotic genes such as P53 in neutrophils. Such effects observed here in our study which need further in depth investigation for clinical implications

In this study, the effect of BM‐MSCs on neutrophils was investigated and it was shown that these cells protect neutrophils from apoptosis through the effect on the expression of pro‐ and anti‐apoptotic genes. In fact, they decreased the expression of pro‐apoptotic gene (P53) and increased the expression of anti‐apoptotic genes (Bcl2 and survivin) in neutrophils which are in line with other studies (Raffaghello et al., 2008; Savill et al., 2002) (Figure 4).

Here, the direct effect of BM‐MSCs on neutrophils by complete contact and their indirect effects by soluble factors in condition medium on neutrophils was assessed. The results confirmed the protective effects of BM‐MSCs on neurophils in both condition. In other words, it seems that the protective effect is mediated through secreted factors by BM‐MSCs and direct contact of BM‐MSCs is not obligatory for their effects on neutrophils. Actually, since the effect on gene expression is observed both in the presence of cells and only their secretions, it appears that the effects of BM‐MSCs are mediated via their secretions and direct physical contact is not necessary (Brandau et al., 2010).

In this regard, it has been shown that incubation of BM‐MSCs supernatant for 24 hr reduces the apoptosis rate in LPS‐stimulated neutrophils by secreting IL‐8 and inhibitory macrophage secretion (Brandau et al., 2010). Neutrophil survival is modulated through survival and death factors, however, their portion in the apoptosis regulation in vivo is not clear (Colotta et al., 1992; Lee et al., 1993; Liles, 1996). It is assumed that BM‐MSCs have the same effect on adult neutrophils by protecting them from cell death in response to external stimulation. In resting neutrophils, BM‐MSCs are able to protect neutrophils from serum‐free cell death. This anti‐apoptotic effect is particularly noticeable in 24‐hr incubation. The minimum time required for the indirect anti‐apoptotic effect shows that the relationship between neutrophils and BM‐MSCs occurs through specific signalling pathways and secretion of specific soluble factors (Ma et al., 1991). The anti‐apoptotic effects of BM‐MSCs were similar to neutrophil incubation with different cytokines, including IL‐1, TNF‐α, IL‐6, granulocyte colony‐stimulating factor (MCSF) and granulocyte macrophage–colony‐stimulating factor GM‐MCSF (Brach et al., 1992; Colotta et al., 1992), which show the importance of secreted factors in paracrine route. Also, it has been reported that the anti‐apoptotic effects of BM‐MSCs is a result of production of IL‐6 cytokine (Rasmusson, 2006).

BM‐MSCs have been reported to inhibit resting and active neutrophils with IL‐8, from apoptosis in normal serum‐supplemented culture media. Human BM‐MSCs reduces the BAX pro‐apoptosis mitochondrial protein via IL‐6 signalling, and increases the anti‐apoptotic mitochondrial protein MCL‐1 and effectively inhibits resting and active neutrophils from apoptosis at ratios up to 500:1 (Raffaghello et al., 2008). Our study was conducted with 1:35 (BM‐MSCs:N) ratio, which was one of the favourable values (Raffaghello et al., 2008). According to studies, the ratio of co‐cultured cells is strongly influenced by their effects, and perhaps carrying out experiments with different proportions would have other results (Raffaghello et al., 2008).

The association between activation of other genes by p53 and apoptosis is due to its ability to control the pro‐apoptotic members of the Bcl_2_ family (Miyashita et al., 1994). Accordingly, p53 causes apoptosis by inducing expression of pro‐apoptotic genes such as BAX or decreasing the expression of anti‐apoptotic genes such as Bcl_2_. In a pathway independent of transcription, p53 activates BAX in the cytoplasm, eventually releasing caspases and causing apoptosis (Allen et al., 1993).

### 4.1 Conclusion

It can be concluded that equine BM‐MSCs and their secreted soluble factors protect neutrophils from apoptosis by decreasing pro‐apoptotic gene expression (p53) and increasing anti‐apoptotic gene expression (Bcl_2_ and survivin) under co‐culture conditions. These finding can be considered in studies which deal with modulation of immune system.

## DECLARATION OF COMPETING INTEREST

The authors declare no potential conflicts of interest.

## AUTHOR CONTRIBUTIONS

AP conceptualized, designed and conducted the study; reviewed and edited the manuscript. JM was involved in designing and conducting the study and analysed the data, and reviewed and edited the manuscript. FS performed the experiment, gathered data and wrote the first draft. MG was involved in performing the experiment. All authors read and approved the final version of the manuscript.
